# Radiotherapy statements of the 18th St. Gallen International Breast Cancer Consensus Conference—a German expert perspective

**DOI:** 10.1007/s00066-024-02209-7

**Published:** 2024-02-23

**Authors:** David Krug, Maggie Banys-Paluchowski, Sara Y. Brucker, Carsten Denkert, Nina Ditsch, Peter A. Fasching, Renate Haidinger, Nadia Harbeck, Jörg Heil, Jens Huober, Christian Jackisch, Wolfgang Janni, Hans-Christian Kolberg, Sibylle Loibl, Diana Lüftner, Marion van Mackelenbergh, Julia C. Radosa, Toralf Reimer, Manfred Welslau, Rachel Würstlein, Michael Untch, Wilfried Budach

**Affiliations:** 1 Klinik für Strahlentherapie, Universitätsklinikum Schleswig-Holstein, Campus Kiel, Arnold-Heller-Straße 3, Haus L, 24105 Kiel, Germany; 2 Klinik für Frauenheilkunde und Geburtshilfe, Brustzentrum, Campus Lübeck, Universitätsklinikum Schleswig-Holstein, Lübeck, Germany; 3 Universitäts-Frauenklinik Tübingen, Tübingen, Germany; 4 Institut für Pathologie, Philipps-Universität Marburg und Universitätsklinikum Marburg (UKGM), Marburg, Germany; 5 Klinik für Frauenheilkunde und Geburtshilfe, Brustzentrum, Universitätsklinikum Augsburg, Augsburg, Germany; 6 Frauenklinik des Universitätsklinikums Erlangen, Comprehensive Cancer Center Erlangen-EMN, Friedrich-Alexander-Universität Erlangen-Nürnberg, Erlangen, Germany; 7 Brustkrebs Deutschland e. V., Hohenbrunn/Munich, Germany; 8 Brustzentrum, Frauenklinik, LMU Klinikum München, Munich, Germany; 9 Brustzentrum Heidelberg, Klinik St. Elisabeth, Heidelberg, Germany; 10 Medizinische Fakultät, Ruprecht-Karls-Universität Heidelberg, Heidelberg, Germany; 11 Brustzentrum, Kantonsspital St. Gallen, St. Gallen, Switzerland; 12 Klinik für Gynäkologie und Geburtshilfe, Sana-Klinikum Offenbach GmbH, Offenbach, Germany; 13 Universitätsfrauenklinik Ulm, Ulm, Germany; 14 Klinik für Gynäkologie und Geburtshilfe, Marienhospital Bottrop gGmbH, Bottrop, Germany; 15 German Breast Group (GBG), Neu-Isenburg, Germany; 16 Centrum für Hämatologie und Onkologie Bethanien, Frankfurt am Main, Germany; 17 Immanuel Klinik Märkische Schweiz, Buckow, Germany; 18 Immanuel Klinik Rüdersdorf, Medizinische Hochschule Brandenburg, Rüdersdorf/Berlin, Germany; 19 Gynäkologie und Geburtshilfe, Campus Kiel, Universitätsklinikum Schleswig-Holstein, Kiel, Germany; 20 Klinik für Gynäkologie, Geburtshilfe und Reproduktionsmedizin, Universitätsklinikum des Saarlandes, Homburg/Saar, Germany; 21 Universitätsfrauenklinik und Poliklinik, Klinikum Südstadt Rostock, Rostock, Germany; 22 Onkologie Aschaffenburg, Hämato-Onkologische Schwerpunktpraxis, Klinikum Aschaffenburg-Alzenau, Aschaffenburg, Germany; 23 Klinik für Gynäkologie und Geburtshilfe, interdisziplinäres Brustzentrum, HELIOS Klinikum Berlin Buch, Berlin, Germany; 24 Klinik für Strahlentherapie und Radioonkologie, Universitätsklinikum Düsseldorf, Düsseldorf, Germany

**Keywords:** Radiation oncology, Ductal carcinoma in situ, Hypofractionation, Breast-conserving surgery, Guideline

## Abstract

**Purpose:**

To summarize the radiotherapy-relevant statements of the 18th St. Gallen Breast Cancer Consensus Conference and interpret the findings in light of German guideline recommendations.

**Methods:**

Statements and voting results from the 18th St. Gallen International Breast Cancer Consensus Conference were collected and analyzed according to their relevance for the radiation oncology community. The voting results were discussed in two hybrid meetings among the authors of this manuscript on March 18 and 19, 2023, in light of the German S3 guideline and the 2023 version of the *Arbeitsgemeinschaft Gynäkologische Onkologie* (AGO) guidelines.

**Results and conclusion:**

There was a high level of agreement between the radiotherapy-related statements of the 18th St. Gallen International Breast Cancer Consensus Conference and the German S3 and AGO guidelines. Discrepancies include the impact of number of lymph node metastases for the indication for postmastectomy radiotherapy.

**Supplementary Information:**

The online version of this article (10.1007/s00066-024-02209-7) contains supplementary material, which is available to authorized users.

## Introduction

The St. Gallen International Breast Cancer Consensus Conference is an interdisciplinary consensus meeting that is held every 2 years [1]. A selected group of experts are invited to participate as active panel members. A list of the complete expert panel can be found in Supplementary Table 1. A list of radiation oncology representatives is shown in Table 1. Prior to the meeting, a voting on questions related to the diagnosis and management of breast cancer is performed. Often, these questions are based on clinical case scenarios. At the meeting, results are presented and discussed. In some cases, the panel voting is supplemented by an audience vote. On a regular basis, results of the St. Gallen International Breast Cancer Consensus Conference are commented on and contextualized by a German expert group [2]. In the past editions, this has mainly focused on questions regarding surgical management and systemic therapy.

**Table 1:** Radiation oncology representatives in the 18th St. Gallen International Breast Cancer Consensus Conference

Name	Country
Charlotte Coles	United Kingdom
Gerd Fastner	Austria
Orit Kaidar-Person	Israel
Icro Meattini	Italy
Lori Pierce	USA
Philip Poortmans	Belgium

In 2023, the conference included radiotherapy issues and, therefore, this publication focusses on radiotherapy-related aspects of the 18th St. Gallen International Breast Cancer Consensus Conference from a German perspective.

## Methods

Statements and voting results from the 18th St. Gallen International Breast Cancer Consensus Conference were collected and analyzed according to their relevance for the radiation oncology community. Radiotherapy-related questions can be found in the following chapters: “Ductal carcinoma in situ,” “Male breast cancer,” “Radiation therapy,” “Axillary surgery,” “Locoregional recurrence after breast-conserving surgery/radiotherapy,” and “Oligometastatic disease.” Percentages were rounded. All questions included the option to abstain from the vote. Results presented are percentages of casted votes. It is important to note that the rate of abstention was relatively high for the radiation-related questions, ranging from 0% (radiotherapy omission for low-risk breast cancer) to 38% (boost irradiation after neoadjuvant chemotherapy). The complete voting results can be found in the online appendix of the consensus publication [1]. The voting results were discussed in two hybrid meetings among the authors of this manuscript on March 18th and 19th, 2023. Results from the 18th St. Gallen International Breast Cancer Consensus Conference are discussed in light of the German S3 guideline [3] and the 2023 version of the *Arbeitsgemeinschaft Gynäkologische Onkologie* (AGO) guidelines [4, 5]. For statements from the AGO guidelines, the Oxford level of evidence (LoE), Oxford grade of recommendation (GR), and AGO grade of recommendation (AGO; ++ = highly beneficial for patients, should be performed; + = limited benefit, can be performed; +/− = may be performed only in individual cases, a general recommendation cannot be given; − = can be of disadvantage for patients and might not be performed; −− = clear disadvantage for patients and should be avoided or omitted in any case) are stated.

## Results

### Ductal carcinoma in situ

Adjuvant radiotherapy for a healthy premenopausal woman with low-risk ductal carcinoma in situ (DCIS; < 2 cm, G1–2, no comedonecrosis) was recommended by 73% of experts while 54% of experts recommended radiotherapy for a postmenopausal patient with the same tumor characteristics. In patients who have endocrine therapy, these numbers decreased to 62 and 40%, respectively. In the German guidelines [3, 6], individual discussion of adjuvant radiotherapy with consideration of the individual risk profile is recommended. Endocrine therapy is not universally recommended in the German guidelines. There is only a minor effect of endocrine therapy on local recurrence (invasive and noninvasive), while the main benefit is reduction of the incidence of contralateral breast cancer. Thus, the decision on adjuvant radiotherapy should be independent of administration of adjuvant endocrine therapy.

Fractionation of adjuvant radiotherapy for DCIS was addressed with two questions using the same case of low-risk DCIS (< 2 cm, G1–2, no comedonecrosis) with either pre- or postmenopausal status. In both situations, moderate hypofractionation was regarded as the preferred treatment (50% for pre- and 34% for postmenopausal) followed by five-fraction partial breast irradiation (PBI; 5% pre- and 20% for postmenopausal), while 0% preferred conventional fractionation for pre- or postmenopausal patients and 20% stated that any of the mentioned treatments are reasonable. While the pivotal randomized controlled trials used conventional fractionation [7], there is growing evidence for moderate hypofractionation. In addition to the numerous randomized controlled trials for invasive breast cancer, the BIG 3–07/TROG 07.01 trial showed similar outcomes among 503 patients randomized to conventional fractionation vs. moderate hypofractionation [8]. The AGO guideline includes moderate hypofractionation for DCIS with the same grade of recommendation as conventional fractionation (LoE 1a, GR A, AGO +) [6]. Although there is less evidence regarding moderate hypofractionation for DCIS than for invasive breast cancer, German experts agree that reduced acute toxicity, increased patient convenience, and decreased costs favor the use of moderate hypofractionation. PBI is considered as a treatment option for patients with low-risk DCIS (LoE 1b, GR B, AGO +; age ≥ 50 years, ≤ 3 cm, G1‑2, R0 [≥ 5 mm], unifocal/unicentric) [6]. Only a minority of PBI trials enrolled patients with DCIS [9–11]. Thus, the optimal fractionation schedule for PBI in patients with DCIS is unclear.

A series of questions presented varying cases of DCIS with differences in age, tumor size, and presence of comedonecrosis. Interestingly, age seemed to be the driving factor for recommendation of adjuvant radiotherapy.

### Male breast cancer

Due to the low incidence of male breast cancer, most recommendations are extrapolated from breast cancer in women. However, 42% of experts recommended conventional mastectomy, 13% nipple-sparing mastectomy, and only 36% lumpectomy with adjuvant radiotherapy. Retrospective population-based data support the use of breast-conserving surgery (BCS) with adjuvant radiotherapy, with similar outcome data compared to mastectomy [12–14]. While the AGO guideline recommends mastectomy as the treatment of choice (LoE 4 GR B AGO ++), the German S3 guideline suggests consideration of breast conservation with adjuvant radiotherapy in patients with a favorable tumor to breast ratio [3].

### Radiation therapy in patients with invasive breast cancer

**Fig. 1:**
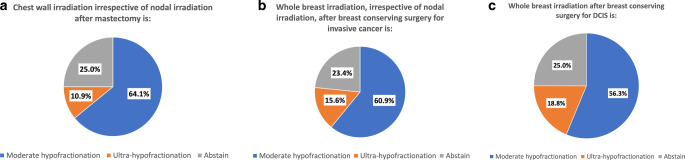
Fractionation of adjuvant radiotherapy in different clinical scenarios: **a** Postmastectomy radiotherapy, **b** whole-breast irradiation for invasive breast cancer, **c** whole-breast irradiation for ductal carcinoma in situ (*DCIS*)

A series of questions addressed the optimal fractionation in different scenarios: postmastectomy radiotherapy (PMRT), whole-breast radiotherapy for invasive breast cancer, both irrespective of regional nodal irradiation (RNI), and whole-breast radiotherapy for DCIS. Moderate hypofractionation was regarded as the preferred option in 64, 61, and 56%, while ultrahypofractionation (5 × 5.2 Gy in 1 week) was chosen by 11, 16, and 19% of experts, respectively. Results are shown in Fig. 1. It is remarkable that conventional fractionation was not listed as a possible answer in any of the scenarios, demonstrating the ongoing paradigm change. While moderate hypofractionation is indisputably the standard of care for whole-breast radiotherapy, there is less evidence for RNI and DCIS. Due to emerging data from randomized controlled trials presented at international conferences demonstrating noninferiority of moderate hypofractionation for RNI, the AGO has upgraded moderate hypofractionation (LoE 1b GR A AGO +) [6]. Although the ESTRO-ACROP consensus guideline [15] states that ultrahypofractionation can be offered as a standard of care or in the context of trials or registries (86.9% consensus), the voting results mentioned above suggest that this is not universally accepted. This is also reflected in the AGO (LoE 1b GR B AGO +/−) and DEGRO guidelines [6, 16]. Nevertheless, German experts agree that ultrahypofractionation is a valid option after BCS or mastectomy without reconstruction if there is no indication for RNI, especially for elderly and frail breast cancer patients who are not considered good candidates for radiotherapy omission. It is somewhat counterintuitive that the recommendation for ultrahypofractionation was highest in DCIS. For a discussion of fractionation in DCIS, please see the paragraph above.

Regarding boost irradiation, one question involved criteria for application and presented four risk factors: age < 50 years, extensive intraductal component, triple-negative or HER2-positive subtype, and G3. Thirty-six percent of experts stated that the presence of one of the mentioned risk factors is sufficient, while 8, 3, and 2% stated that two, three, or four factors should be present, respectively. Other boost criteria are used by 17% of experts. In the German guidelines [3, 6], one of the abovementioned criteria is sufficient for boost irradiation. In patients with pathologic complete response (pCR), 12.6% would omit the boost, 23.8% would omit it only in patients with clinical stage I–II, and 25.4% would not recommend omission of boost irradiation in the context of pCR. In the absence of clinical data regarding this question, German experts recommend evaluating this on a case-by-case basis with consideration of the individual risk profile.

**Fig. 2:**
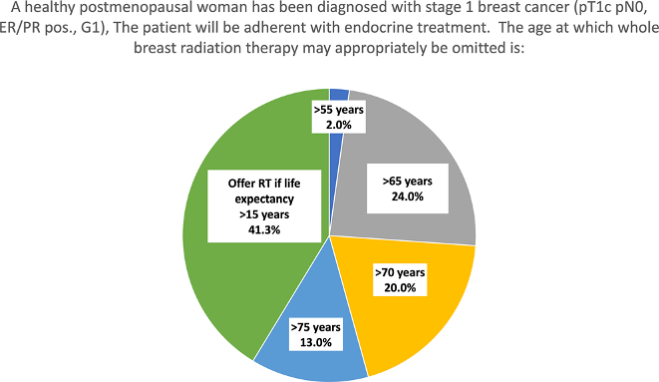
Thresholds for omission of whole-breast irradiation after breast-conserving surgery for patients with low-risk breast cancer. *ER* estrogen receptor, *PR* progesteron receptor, *RT* radiotherapy

For an otherwise healthy postmenopausal woman with low-risk breast cancer (pT1b pN0, G1, strong expression of estrogen [ER] and progesterone receptors, presumed adherence to endocrine therapy), experts were asked to state at which age they would consider omission of whole-breast radiotherapy appropriate. Results are shown in Fig. 2. The largest group of experts (41%) chose to offer radiotherapy if life expectancy is > 15 years. In the audience vote, 39% chose life expectancy > 15 years, while 23 and 13% chose age > 70 years and age > 75 years, respectively. Regarding the outcome of the PRIME-II trial [17], 64% answered that their main takeaway from the trial was that radiotherapy lowers the risk of local recurrence and is therefore effective, while 27% answered that it does not alter survival and can therefore be omitted. Since several randomized controlled trials including the PRIME-II trial demonstrated increasing benefit of adjuvant radiotherapy regarding prevention of local recurrence with longer follow-up [17–19], and because life expectancy is steadily increasing, the German guidelines recommend consideration of radiotherapy omission only in patients with a presumed life expectancy < 10 years [3, 6].

**Fig. 3:**
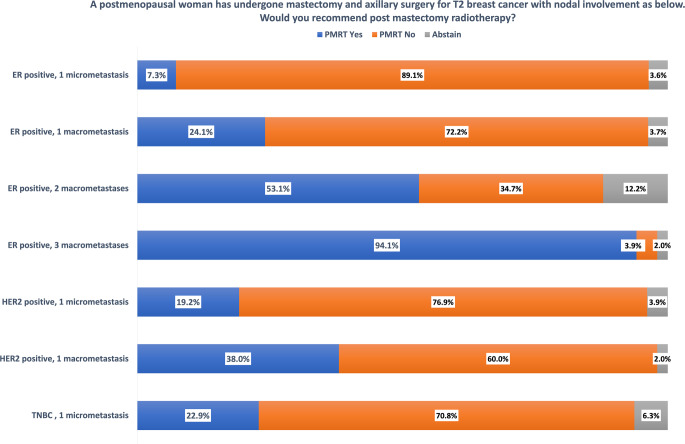
Postmastectomy radiotherapy (*PMRT*) according to the disease burden in the axilla. *ER* estrogen receptor, *HER2* humen epidermal growth factor receptor 2, *TNBC* triple negative breast cancer

PMRT was analyzed with a series of questions presenting the same case (postmenopausal woman with a pT2 tumor with ER positivity) with an increasing tumor load in the lymph nodes. With an increase in the nodal stage, the percentage of experts recommending PMRT steadily increased (Fig. 3). For a postmenopausal patient with ER-positive pT3 breast cancer, 49% voted for and 45.6% against PMRT. In a postmenopausal patient with pT2 breast cancer and one micrometastasis, 38% would recommend PMRT if HER2 positive and 23% if triple negative. In the German guidelines [3, 6], the indication for PMRT in patients with 1–3 macrometastases is based on grading, biologic subtype, and lymphovascular invasion, while the number of involved lymph nodes is not considered a major decision criterion for patients with stage pN1a. There is no specific recommendation for PMRT in patients with micrometastatic lymph node involvement. In an ad hoc vote, the expert panel was equally divided on whether implant-based reconstruction should influence the decision for or against PMRT. The German expert panel states that if patient selection and surgical technique of breast reconstruction are adequate, the oncological outcome is similar to standard mastectomy. If there is an indication for PMRT based on clinical risk factors, it should not be omitted solely because of implant reconstruction.

A heterozygous mutation in the *ATM* gene is considered a contraindication to radiotherapy by 21% of experts, while 74% stated that it should not be considered a contraindication. This scenario is not mentioned in the German guidelines. The German Consortium for Hereditary Breast and Ovarian Cancer similarly states that a heterozygous mutation in the ATM gene should not be considered a contraindication to radiotherapy [20]. However, a mutation in the *TP53* gene would typically be considered a contraindication to radiotherapy, and mastectomy should be preferred even in early disease stages [21].

### Axillary surgery

In a patient with triple-negative breast cancer who undergoes primary systemic therapy, with residual disease in the axilla during surgery and a macrometastasis in one out of three sentinel lymph nodes, 47% of experts recommended completion axillary lymph node dissection (ALND), 20% recommended axillary radiotherapy, and 29% recommended both modalities. Results were similar in the audience vote. The recommendation for ALND in this setting is in accordance with the German guidelines [3, 6, 22]. However, axillary radiotherapy after ALND is only recommended if there is macroscopic residual tumor in the axilla after surgery (LoE 5 GR D AGO ++) [22].

### Locoregional recurrence after breast-conserving surgery/radiotherapy

Regarding the management of local recurrence after prior BCS with adjuvant radiotherapy, the case of a 63-year-old woman with ER-positive cT1 breast cancer amenable to repeat BCS and with minor toxicity from prior radiotherapy was presented. If the patient presented 9 years after primary breast cancer, 58% of experts would recommend repeat BCS with adjuvant radiotherapy, 26% mastectomy, and 15% BCS without radiotherapy. If the local recurrence occurred 3 years after primary breast cancer, 74% of experts would prefer mastectomy and only 19 and 6% would offer BCS with or without radiotherapy. In the AGO guidelines, repeat BCS with PBI is considered an alternative to mastectomy (LoE 2b GR B AGO +). Importantly, a radiation oncologist should be consulted before surgery to evaluate the suitability of repeat irradiation, since BCS without additional radiotherapy results in an increased risk of local recurrence (LoE 2b GR B AGO +/−).

### Oligometastatic disease

In a woman with cT2 cN+ ER-negative HER2-positive breast cancer with a solitary lung metastasis and clinical complete response to first-line systemic therapy, 68% of experts recommended surgery of the primary tumor with adjuvant radiotherapy, while 14% recommended neither surgery nor radiotherapy. If the tumor were triple-negative, only 63% would perform surgery and adjuvant radiotherapy and 20% would perform neither surgery nor radiotherapy. In a patient with ER-positive HER2-negative breast cancer, the results were similar to triple-negative disease. In the AGO guidelines, local treatment of the primary tumor is not recommended (LoE 1b GR B AGO −) in patients with visceral metastases [5]. However, the abovementioned individual scenario with a clinical complete response of a solitary metastasis is not adequately covered by clinical trials. Hence, local therapy may be discussed on an individual basis. In a patient with stage II breast cancer and an isolated contralateral axillary lymph node metastasis, 75% of the expert panel recommended definitive treatment with curative intent including contralateral axillary surgery and radiotherapy, while 16% recommended against it. This result is in line with the AGO guidelines [5].

## Conclusion

There was a high level of agreement between the radiotherapy-related statements of the 18th St. Gallen International Breast Cancer Consensus Conference and the German S3 and AGO guidelines. Moderate hypofractionation is considered standard of care for PMRT, RNI, and whole-breast irradiation in patients with invasive breast cancer and DCIS. In the next guideline update, it may be considered to incorporate the number of lymph node metastases in the decision-making process for PMRT, since this seemed to be a major driver in the decision process. However, there are no prospective data to support this.

### Supplementary Information

Supplementary Table 1: Complete list of representatives in the 18th St. Gallen International Breast Cancer Consensus Conference
