# Supplementary material for: Radiotherapy statements of the 18th St. Gallen International Breast Cancer Consensus Conference—a German expert perspective
Source: Strahlenther Onkol. 2024 Feb 23;200(6):461–7. doi: 10.1007/s00066-024-02209-7 (PMC11111516; doi:10.1007/s00066-024-02209-7)
Supplement: Supplementary file 1 — Supplementary Table 1: Complete list of representatives in the 18th St. Gallen International Breast Cancer Consensus Conference [file 66_2024_2209_MOESM1_ESM.docx]

Supplementary Table 1: Complete list of representatives in the 18^th^ St. Gallen International Breast Cancer Consensus Conference

| **Name** | **Country** |
| --- | --- |
| Stephan Aebi | Switzerland |
| Meteb Al-Foheidi | Saudi Arabia |
| Fabrice André | France |
| Mikola Anikusko | Ukraine |
| Rajendra Badwe | India |
| Andrea V. Barrio | USA |
| Carlos Barrios | Brazil |
| Jonas Bergh | Sweden |
| Hervé Bonnefoi | France |
| Denisse Bretel Morales | Peru |
| Sara Brucker | Germany |
| Harold J. Burstein | USA |
| Carlos Caldas | UK |
| David Cameron | UK |
| Fatima Cardoso | Portugal |
| Maria Joao Cardoso | Portugal |
| Lisa Carey | USA |
| Steven Chia | Canada |
| Charlotte Coles | UK |
| Javier Cortes | Spain |
| Giuseppe Curigliano | Italy |
| Jana de Boniface | Sweden |
| Suzette Delaloge | France |
| Angela DeMichele | USA |
| Carsten Denkert | Germany |
| Gerd Fastner | Austria |
| Florian Fitzal | Austria |
| Prudence Francis | Australia |
| Heba Gamal | Egypt |
| Oreste Gentilini | Italy |
| Michael Gnant | Austria |
| William Gradishar | USA |
| Bahadir Gulluoglu | Turkey |
| Nadia Harbeck | Germany |
| Jörg Heil | Germany |
| Chiun-Sheng Huang | Taiwan |
| Jens Huober | Switzerland |
| Zefei Jiang | China |
| Orit Kaidar-Person | Israel |
| Marleen Kok | Netherlands |
| Eun-Sook Lee | Korea |
| Sherene Loi | Australia |
| Sibylle Loibl | Germany |
| Miguel Martin | Spain |
| Icro Meattini | Italy |
| Monica Morrow | USA |
| Ann Partridge | USA |
| Frederique Penault-Llorca | France |
| Martine Piccart | Belgium |
| Lori Pierce | USA |
| Philip Poortmans | Belgium |
| Meredith Regan | USA |
| Jorge Reis-Filho | USA |
| Isabel Rubio | Spain |
| Hope Rugo | USA |
| Emiel Rutgers | Netherlands |
| Cristina Saura | Spain |
| Elzbieta Senkus | Poland |
| Zhiming Shao | China |
| Christian Singer | Austria |
| Tanja Spanic | Slovenia |
| Beat Thuerlimann | Switzerland |
| Masakazu Toi | Japan |
| Sara Tolaney | USA |
| Nicholas Turner | UK |
| Andrew Tutt | UK |
| Marie-Jeanne Vrancken Peeters | Netherlands |
| Toru Watanabe | Japan |
| Walter Weber | Switzerland |
| Hans Wildiers | Belgium |
| Binghe Xu | China |
